# Natural Fibers: Why Are They Still the Missing Thread in the Textile Fiber Pollution Story?

**DOI:** 10.1021/acs.est.4c05126

**Published:** 2024-07-10

**Authors:** Thomas Stanton, Alana James, Miranda T. Prendergast-Miller, Anne Peirson-Smith, Chimdia KeChi-Okafor, Matteo D. Gallidabino, Anil Namdeo, Kelly J. Sheridan

**Affiliations:** † Department of Geography and Environment, Loughborough University, Loughborough LE11 3TU, U.K.; ‡ Department of Design, Northumbria University, Newcastle NE1 8ST, U.K.; § Department of Geography and Environmental Sciences, Faculty of Engineering and Environment, Northumbria University, Newcastle NE1 8ST, U.K.; ∥ King’s Forensics, Department of Analytical, Environmental and Forensic Sciences, King’s College London, London SE1 9NH, U.K.; ⊥ Centre for Forensic Science, Department of Applied Sciences, Faculty of Health and Life Sciences, Northumbria University, Newcastle NE1 8ST, U.K.; # The Microfibre Consortium, Newminster House, 27−29 Baldwin Street, Bristol BS1 1LT, U.K.

**Keywords:** natural fibers, textile fiber pollution, textile fiber populations

In 2015, Ladewig et al.^1^ suggested for the first time that natural textile fibers (hereafter natural fibers), such as cotton and wool, be considered as pollutants in their own right, alongside microplastic fibers such as polyester and acrylic. Here we define natural textile fibers as biological materials that have been modified for textile applications, excluding fibrous materials derived from the breakdown of plant and animal materials (e.g., leaves and animal hair). Ladewig et al. primarily focused on the potential for natural fibers to act as a source of various polluting chemicals associated with fiber production as they degrade in the environment.^1^

In the years that have followed, environmental science research has begun to acknowledge and semiquantify the presence of natural fibers in their environmental samples.^2^ The first full textile fiber population assessment in the environmental sciences was published in 2019.^2^ Its finding that, when they are looked for, natural textile fibers are more prevalent than plastic fibers has subsequently been repeated across environmental matrices and around the world. These studies have repeatedly found that natural fibers account for >70% of all textile fibers recovered.^2,3^ However, natural textile fibers are not regularly looked for.

However, although the environmental science literature has quantified entire textile fiber populations for just five years (Figure 1), this is established knowledge in other disciplines. In the forensic sciences, for example, the environmental prevalence of natural fibers has been studied for decades.^4^ Here, we implore the scholarly community to continue to develop and diversify natural fiber research.

**Figure 1:**
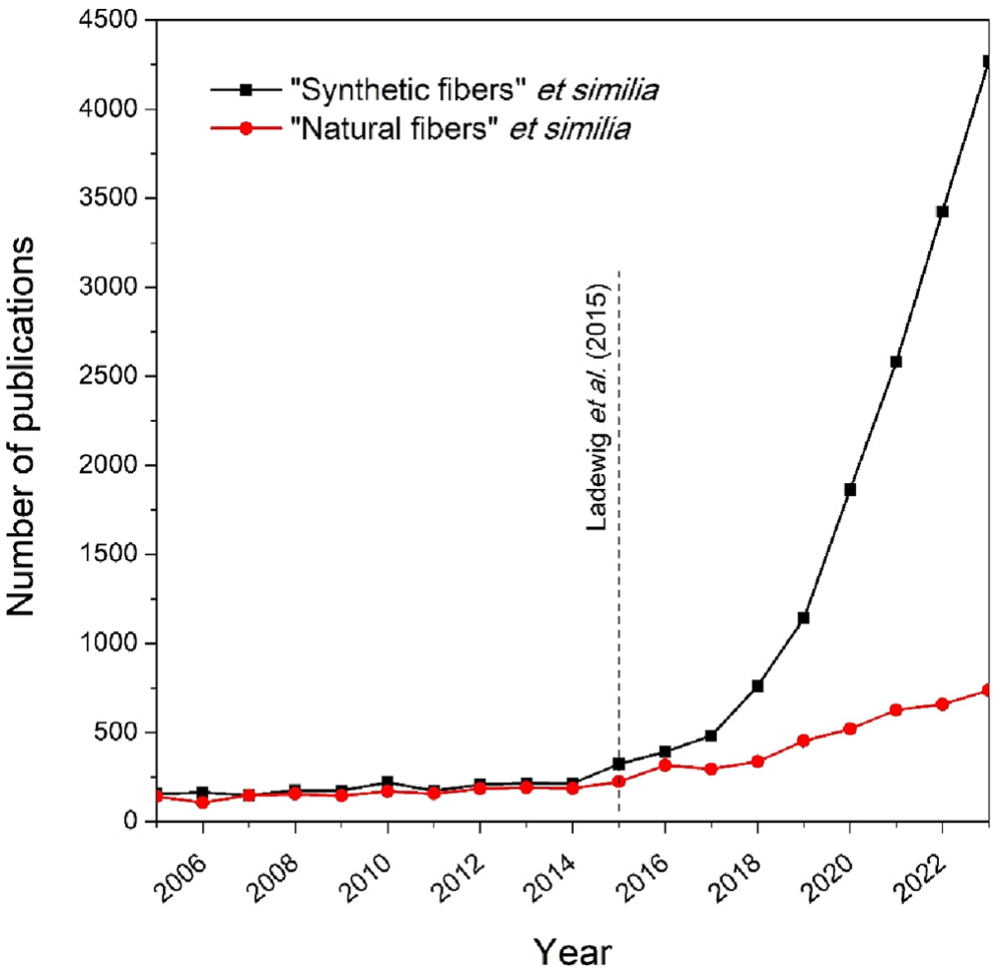
Number of publications involving the study of synthetic and natural fibers across environmental sciences. Literature searching was performed with keywords through Elsevier’s Scopus (April 9, 2024) and outputs limited to the “environmental sciences” subject area only. The keywords retained for each category were as follows: “Synthetic fibres” et similia: “microplastic”, “micro-plastic”, “synthetic fibre”, “synthetic fiber”, “plastic fibre” and “plastic fiber”. “Natural fibres” et similia: “natural fibre” and “natural fiber”. Keywords were searched within the article title, abstract, and paper keywords.

## Need for Unified and Accurate Terminology

Textile fibers fall into three broad categories: synthetic, natural, and regenerated.^2^ Synthetic fibers made from plastic polymers, whether derived from petrochemical sources or agricultural crops, have been the primary focus of fiber pollution research. Their definition is relatively simple. Whether obtained from fossil fuel or biomass sources, synthetic fibers such as polyester and acrylic are formed from plastic polymers.

Non-plastic textile fibers, derived from plant and animal sources, fall into two categories: fibers derived directly from their plant or animal source, such as cotton and wool (natural fibers), and those that are the product of specific chemical treatments and reconstitutions of natural polymers, particularly cellulose, such as viscose and modal (known as semisynthetic or regenerated fibers).

While these definitions are established in the textile industry,^2^ their use in the environmental science literature has been confused, particularly for regenerated fibers. Regenerated fibers have also been mis-categorized as plastic, natural, and biobased fibers within the published literature (e.g., ref (5) and references therein). It is vital that environmental scholars accurately describe their fibers of interest using these established definitions, as defined by the textile industry, to support transparent knowledge exchange.

## Current Natural Fiber Research Gaps: Persistence, Toxicity, and Chemical Load

Misconceptions associated with the biodegradability of natural fibers, and a narrative focus on the fiber itself over the chemical load associated with its manufacture, have resulted in perhaps this field’s most dangerous assumption: that natural textile fibers biodegrade to harmless constituents. While it is true that natural polymers are more biodegradable than synthetic polymers, specific modifications to some natural fibers, which enhance their suitability for textile applications, in fact alter their polymeric structure. Mercerization of cotton, for example, transforms cellulose I, readily biodegradable and widely found in the environment, into cellulose II, a more stable polymer, not commonly found in the environment. Moreover, while the leaching of chemicals associated with natural fiber production, e.g., chemical colorants and finishes, was the primary concern of Ladewig et al.,^1^ these chemical additions can also further reduce the rate of biodegradation of natural textile fibers.

Beyond their presence in the environment, natural fibers are also an emerging focus of ecotoxicological studies. For example, Siddiqui et al.^5^ report that exposure to cotton textile fibers influences both the behavior and the growth of invertebrate (mysid shrimp) and fish (inland silverside) test organisms and find that this varies with salinity.

However, despite this small and emerging body of work, the potential risks associated with natural fibers continue to be largely overlooked by science, industry, and legislators, and nine years since it was first suggested, the chemical impacts of natural textile fibers are yet to be considered at scale.^1^

## The Way Forward: Multi-, Inter-, and Transdisciplinarity

Though our understanding of natural textile fiber ubiquity, pathways, and impacts continues to lag far behind that of plastic fibers, where it lags most is the acknowledgment and recognition of these anthropogenic particles as an environmental problem. This awareness gap spans the scientific, public, industrial, and legislative sectors, despite the work that has been undertaken since 2015. Addressing this gap is key to accelerating the necessary comprehensive social and environmental impact assessments of all textile fiber types, but it cannot be achieved from the environmental science perspective alone.

The environmental impacts of the fashion and textile industry are notoriously difficult to quantify due to the scale of the industry’s globalized supply chains. Establishing a complete understanding of this industry’s global environmental footprint, and communicating it effectively, therefore has considerable potential in advancing, and clarifying, the public understanding of sustainability.

However, at present the fiber story of this environmental footprint remains half told and relies on unqualified assumptions, potentially driven by greenwashing manifestos or industry lobbying, that textiles and garments made from natural fibers must be more sustainable and better for the environment. Responding to this gap in our textile fiber understanding necessitates further collaborations with scholars from multiple disciplines, including the design and the creative industries, to ensure that future research considers the position of natural fibers within social, economic, industrial, and legislative landscapes.

## Why Are Natural Fibers Still the Missing Thread in the Textile Fiber Pollution Story?

Nearly a decade after Ladewig et al.^1^ first identified natural fibers as a potential missing link in chemical pollution, we propose that natural fibers in fact represent a missing thread in sustainable textile debates. Textile fiber pollution represents perhaps society’s most tangible connection to its environmental footprint. We interact with these anthropogenic particles perhaps more than any other particle type. A complete textile fiber pollution story therefore has considerable potential in broader sustainability narratives. Therefore, we urge those who wish to advance our understanding of textile fiber pollution to consider fibers of all types in their work.

Progress in this space requires that all three of the topics introduced here be addressed. Inaccurate and imprecise terminology confuses interpretations of published work and limits the generation, and hinders the sharing, of new knowledge. Assumptions of biodegradability and omissions of industry-dominating textile fiber types such as cotton and wool from chemical and ecotoxicology research facilitate greenwashing and compound historic, untested assumptions of relative harm. Environmentally relevant natural textile fiber research is also established beyond the environmental sciences. The environmental science community must work with these complementary disciplines, and the tools they use, to fill the research gaps identified here and advance our understanding of the social and environmental contexts of textile fiber pollution in its entirety.

Until this is done, natural fibers will remain missing threads in the textile fiber pollution story. In 2015, three key priorities for natural fiber research were identified:^1^ understanding the pathways and prevalence of natural fibers, establishing natural fiber toxicity, and determining the chemical impacts of natural fibers. Here we reiterate the need for these questions to be explored at a rate greater than that at which they have been over the past nine years.

However, debates about the relative environmental impacts of natural and plastic fibers in the fashion and textiles industry, and the disproportionate focus on plastic materials, extend beyond the environmental fate of fibers alone. In recognition of this, and in light of the natural fiber research published since 2015, we identify three further lines of inquiry, which must be addressed beyond the environmental sciences for the potential to inform sustainability narratives identified here to be realized:

(1) Accurate assessments of the extent of the textile industry’s environmental impact, informed by evidence-based and representative environmental research findings for all fiber types, and transparent communication of these scientific data
(2) Integration of these data into the design and development of new materials, products, and processes and throughout all stages of textile product development
(3) Reflective research and industry action informed by emerging policy and legislation (e.g., the Product Environmental Footprint and the Global Plastics Treaty) to identify and clarify responsibilities for relevant parties from across the fashion and textile value chain

Continued promotion of natural fibers as greener alternatives to plastic pollution by scientists, industry, and legislators, based primarily on the materials they are not, risks misleading consumers and misdirecting sustainability initiatives. Current applications of the precautionary principle to textile fiber use, which facilitate business-as-usual consumption through material substitution, must therefore be challenged. This challenge must recognize and expand the growing literature base that demonstrates that dyed, treated, and mass-produced natural textile fibers are, in fact, inherently unnatural.^2^
